# Conservative Management of Thrombotic Microangiopathy in a Renal Transplant Recipient: The Importance of Early Recognition

**DOI:** 10.1002/ccr3.70351

**Published:** 2025-03-27

**Authors:** Ahmad S. Matarneh, Sundus Sardar, Abdel‐rauof Akkari, Pankhuri Mohan, Naman Trivedi, Gurwant Kaur, Nasrollah Ghahramani

**Affiliations:** ^1^ Department of Nephrology Pennsylvania State Milton S Hershey Medical Center Hershey Pennsylvania USA; ^2^ Department of Medicine‐Pediatrics Pennsylvania State Milton S Hershey Medical Center Hershey Pennsylvania USA

**Keywords:** allograft dysfunction, calcinurin inhibitors, ESRD, immunosuppressed, renal transplant, TMA

## Abstract

Thrombotic microangiopathy (TMA) is a rare but severe complication in renal transplant recipients, often linked to tacrolimus. Early recognition and intervention, such as stopping the drug, are crucial to preventing graft loss. This case highlights the importance of prompt diagnosis and vigilance for subtle signs to ensure effective management.

## 1 Introduction

Kidney transplantation is a lifesaving option for end‐stage kidney disease (ESKD). However, lifelong immunosuppressive therapy, necessary to prevent rejection and preserve graft function, carries inherent risks [1]. Calcineurin inhibitors (CNIs) like tacrolimus and cyclosporine are essential in immunosuppressive regimens for kidney transplantation [2]. Despite their efficacy, CNIs have significant side effects, including nephrotoxicity and, critically, thrombotic microangiopathy (TMA) [3]. Thrombotic microangiopathy is a severe complication involving microangiopathic hemolytic anemia, thrombocytopenia, and renal injury. Its pathogenesis often involves endothelial injury and thrombus formation, potentially leading to irreversible graft loss and death [4]. Thrombotic microangiopathy symptoms often overlap with other transplant‐related complications, making diagnosis challenging [5]. Prompt recognition is key to halting disease progression, with early intervention potentially averting severe graft dysfunction. This case highlights the importance of timely detection, particularly in patients with recent rejection episodes and fluctuating immunosuppressive drug levels.

## 2 Case History and Examination

A 41‐year‐old male with a medical history significant for adult polycystic kidney disease, which had progressively worsened to end‐stage kidney disease (ESKD), underwent a deceased donor kidney transplant 6 years prior. Following the transplant, he was maintained on an immunosuppressive regimen that included mycophenolate mofetil and extended‐release tacrolimus, tailored to prevent rejection and maintain optimal graft function. Despite this regimen, recent clinical evaluations showed a progressive decline in renal function over several weeks, with serum creatinine levels rising from a baseline of 2.2–3.5 mg/dL.

Given the increasing creatinine and concerns for graft health, a kidney biopsy was performed 2 weeks prior to his current presentation. The biopsy results revealed signs of acute T‐cell‐mediated rejection (Grade 1A) and chronic active antibody‐mediated rejection (ABMR) with severe transplant glomerulopathy, moderate interstitial fibrosis, tubular atrophy, and arterial intimal fibrosis, along with moderate tubulointerstitial inflammation, concurrent chronic active ABMR, pointing to a dual rejection process. In response, his immunosuppressive therapy was intensified with the addition of high‐dose intravenous steroids and intravenous immunoglobulin (IVIG) to control the immune response and mitigate further graft damage. His tacrolimus dose was increased following the findings of rejection.

However, despite these therapeutic adjustments, the patient presented to the emergency department with new and concerning symptoms: unexplained bruising, diffuse abdominal pain, persistent nausea, and episodes of diarrhea. These symptoms raised red flags for a potentially severe complication, especially in light of his recent rejection episodes, ongoing immunosuppressive therapy, and increasing creatinine.

## 3 Differential Diagnosis, Investigations, and Treatment

On admission, laboratory tests revealed a significant drop in platelet count from 130 to 35 K/μL, indicating marked thrombocytopenia. Additionally, there was a worsening of anemia, with hemoglobin levels falling from 8.9 to 7.1 g/dL. Notably, serum creatinine had continued to rise, now measuring 5.25 mg/dL, indicating a considerable decline in renal function and raising further concern for potential graft failure. This clinical picture, combined with his complex rejection history and elevated tacrolimus levels, led to a high suspicion of TMA, a potentially life‐threatening complication in transplant recipients.

**FIGURE 1:**
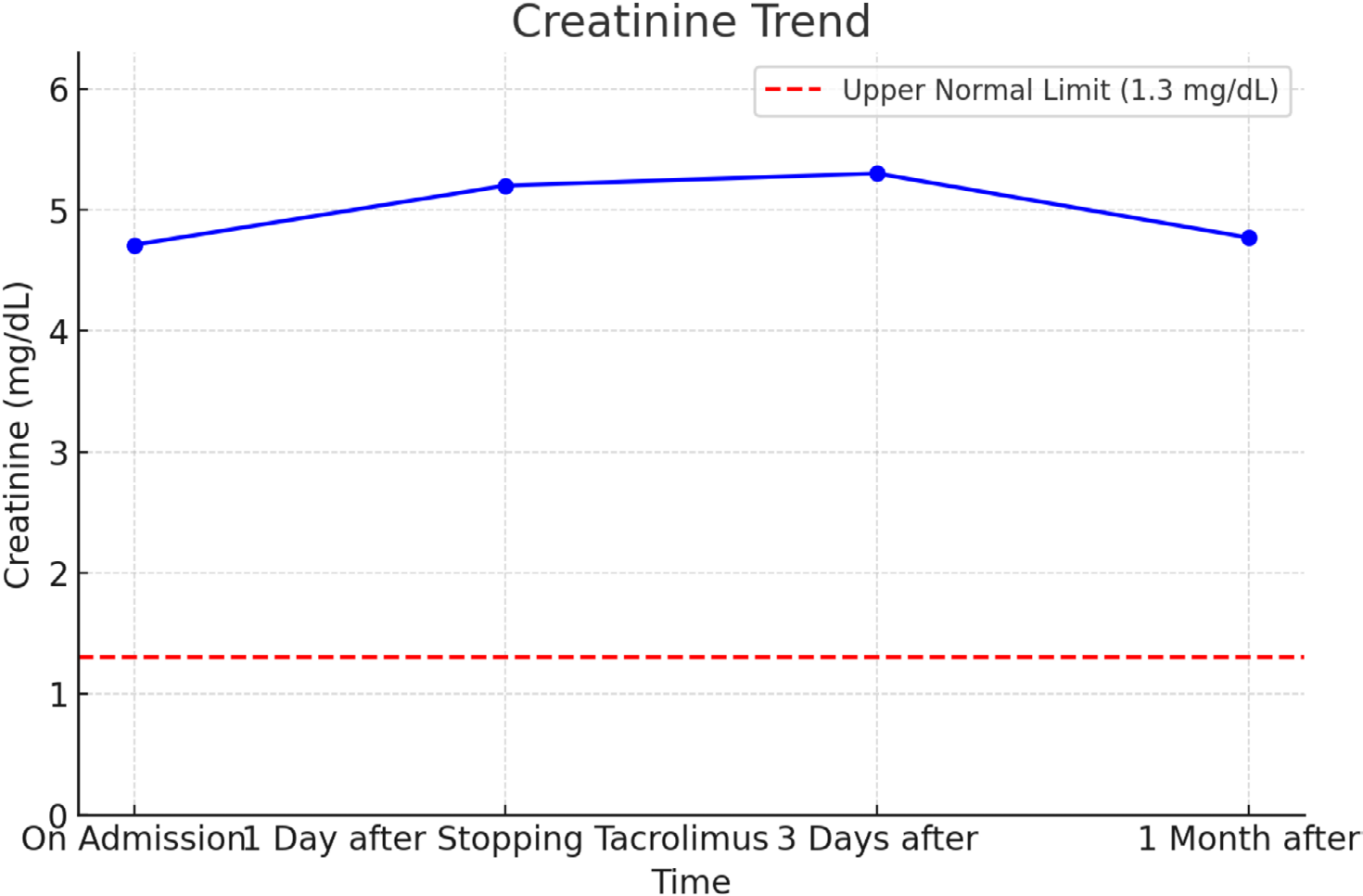
Creatinine trend over time.

To manage this suspected TMA, the patient was promptly transferred to our center for specialized care and further diagnostic evaluation. Additional laboratory tests at our facility provided further insights: lactate dehydrogenase (LDH) levels were markedly elevated at 5727 U/L, and haptoglobin levels were notably low (< 10 mg/dL), both of which are indicative of ongoing hemolysis. A peripheral blood smear revealed the presence of schistocytes, further supporting the diagnosis of hemolysis. To rule out alternative etiologies, an extended enteric pathogen panel was done, and it was negative for any bacteria that might explain TMA; a total bilirubin test was also performed, showing a normal level of 0.5 mg/dL, which effectively ruled out hepatic causes for the hemolysis Table 1. Figure 1 shows creatinine trends during and shortly after hospitalization.

**TABLE 1:** Laboratory evaluation.

Laboratory value	Reference range	On admission	1 day after stopping tacrolimus	3 days after	1 month after
Hemoglobin	13–17 g/dL	6.3	7.1	8.4	11.1
White blood cells	4–10 k/μL	5.6	4.97	5.8	4.7
Tacrolimus level	4–6 ng/mL	10.1	5.4	—	—
Platelets	150–300 k/μL	28	36	64	187
Creatinine	0.7–1.3 mg/dL	4.71	5.2	5.3	4.77
BUN	6–23 mg/dL	74	72	67	64
Potassium	3.5–5.1 mmol/L	5.8	4.6	3.5	4.1
Sodium	136–145 mmol/L	142	137	139	135
Haptoglobin	30–200 mg/dL	< 10	—	—	—
LDH	135–250 μ/L	527	—	—	—
ALT	0–41 μ/L	26	15	11	19
AST	0–40 μ/L	16	26	21	15

Abbreviations: ALT, alanine aminotransferase; AST, aspartate aminotransferase; BUN, blood urea nitrogen; LDH, lactate dehydrogensae.

A comprehensive diagnostic workup ensued, including an ADAMTS13 activity assay, which returned normal, effectively excluding thrombotic thrombocytopenic purpura (TTP) as a differential diagnosis. Additionally, a Coombs test was negative, ruling out autoimmune hemolysis. These findings collectively confirmed that the patient's clinical picture was consistent with tacrolimus‐induced TMA, a rare but serious complication associated with CNI's in transplant recipients.

Due to severely low platelet counts, a follow‐up kidney biopsy was not feasible, as the risks of bleeding outweighed the potential diagnostic benefits. Given the high clinical suspicion of TMA secondary to tacrolimus toxicity, an immediate change in the patient's immunosuppressive regimen was deemed essential. Tacrolimus was discontinued and replaced with belatacept, a co‐stimulatory inhibitor with a safer option for management of this profile in terms of endothelial toxicity, thereby reducing the risk of further microvascular injury. Belatacept was considered an appropriate alternative, as the patient tested seropositive for Epstein–Barr virus (EBV) via polymerase chain reaction (PCR), meeting the safety requirements for belatacept use in transplant patients.

## 4 Outcome and Follow‐Up

Following this immunosuppressive adjustment, the patient's clinical course stabilized. He showed a gradual improvement in platelet counts and hemoglobin levels, along with a stabilization in kidney function. He was discharged from the hospital with instructions to continue belatacept and other maintenance immunosuppressive therapy. Follow‐up assessments confirmed stable renal function with serum creatinine within acceptable ranges around 4 mg/dL, as well as normalized platelet counts (154 K/μL) and improved hemoglobin (11.5 g/dL) indicating successful management of the TMA.

## 5 Discussion

Thrombotic microangiopathy is a severe and potentially life‐threatening complication in renal transplant recipients [6]. It necessitates prompt recognition and timely intervention to prevent irreversible damage to the transplanted kidney, as well as the potential for systemic complications [7]. Thrombotic microangiopathy in this patient population is often induced by CNIs, which are crucial immunosuppressive agents but carry a significant risk of endothelial injury when used at high doses or when trough levels are elevated [8]. This endothelial damage initiates a cascade involving platelet activation, thrombus formation, and subsequent microangiopathy in the renal microvasculature. Over time, these thrombi obstruct the blood supply within the kidney, leading to tissue ischemia and damage, which, if unchecked, can progress to graft failure [9].

High‐dose tacrolimus, a frequently prescribed CNI, has been particularly associated with TMA due to its dose‐dependent toxicity and impact on endothelial cell function. Tacrolimus toxicity manifests as endothelial cell dysfunction, which heightens the risk for TMA [10]. In addition to direct CNI‐related toxicity, other factors contribute to TMA pathogenesis in kidney transplant patients, including immune‐mediated mechanisms such as ABMR, infections, and preexisting genetic susceptibilities [11]. In this case, the patient's recent episode of ABMR likely predisposed him to TMA, as immune activation and endothelial damage from ABMR contribute to the development of thrombotic microangiopathy. The dual impact of ABMR and elevated tacrolimus levels created a perfect setting for endothelial injury, ultimately triggering TMA.

The role of infections, particularly viral infections such as hepatitis C, HIV, and BK virus, in TMA development has been well documented. These infections can provoke immune responses that exacerbate endothelial injury, promoting a similar pathological process as seen with CNI toxicity [12]. Viral infections are known to trigger immune‐mediated endothelial injury and platelet aggregation, further compounding the risk of TMA in transplant recipients who are already immunocompromised [13]. In this case, although viral infections were ruled out as direct contributors, the elevated tacrolimus levels in conjunction with ABMR created a high‐risk environment for endothelial damage and subsequent TMA development.

Diagnosing TMA in kidney transplant recipients is notably challenging due to its nonspecific and often subtle early clinical signs. Common symptoms—such as bruising, abdominal pain, nausea, and diarrhea—can be mistakenly attributed to other, more benign posttransplant complications, such as minor infections or medication side effects [12]. Anemia and thrombocytopenia, both common findings in transplant patients, further obscure the diagnosis of TMA [14]. Anemia may be misattributed to chronic kidney disease (CKD) or immunosuppressive medications, whereas thrombocytopenia may be thought to result from the use of certain drugs or immune suppression. Consequently, clinicians must maintain a high index of suspicion, especially in cases where there is a history of ABMR or elevated CNI levels, as these factors increase the risk of TMA.

In this patient, laboratory investigations were crucial to confirming the diagnosis. The markedly elevated LDH levels, low haptoglobin, and the presence of schistocytes on a peripheral blood smear provided strong indicators of hemolysis, a hallmark of TMA. A normal ADAMTS13 activity level excluded TTP, whereas a negative Coombs test ruled out autoimmune hemolysis. These findings, together with the patient's clinical picture and elevated tacrolimus levels, were consistent with tacrolimus‐induced TMA.

Once TMA is suspected, the immediate discontinuation of the offending drug is paramount to halting disease progression [15]. In this case, tacrolimus was replaced with belatacept, an immunosuppressant with a favorable profile regarding endothelial toxicity. Belatacept functions as a co‐stimulatory inhibitor and is associated with a lower risk of endothelial damage, making it a safer alternative for patients with suspected CNI‐induced TMA. This therapeutic adjustment was effective, as demonstrated by the patient's clinical improvement—specifically, increased platelet counts, stabilized hemoglobin levels, and improved renal function.

In conclusion, this case underscores the critical importance of early recognition and management of TMA in kidney transplant recipients. Although initial signs of TMA are often subtle and nonspecific, a high degree of clinical suspicion and prompt intervention can prevent further complications and avoid graft loss. Strategies that enhance early detection and enable timely treatment adjustments are essential in managing TMA and improving patient outcomes.

Although TMA remains a rare yet severe complication in kidney transplantation, this case illustrates the potential for favorable outcomes when it is promptly diagnosed and managed. Vigilant posttransplant monitoring and timely intervention in response to early signs of TMA can significantly reduce the risk of graft failure, ultimately leading to improved survival rates and quality of life for transplant recipients.

## Author Contributions

**Ahmad S. Matarneh:** conceptualization. **Sundus Sardar:** data curation, investigation, writing – original draft, writing – review and editing. **Abdel‐rauof Akkari:** conceptualization, writing – original draft, writing – review and editing. **Pankhuri Mohan:** writing – original draft, writing – review and editing. **Naman Trivedi:** conceptualization, writing – original draft, writing – review and editing. **Gurwant Kaur:** conceptualization, writing – original draft, writing – review and editing. **Nasrollah Ghahramani:** conceptualization, writing – original draft, writing – review and editing.

## Ethics Statement

Ethical approval for this study was waived by penn‐state ethical committee because this is a case report.

## Consent

Written informed consent was obtained from the patient to publish this case.

## Conflicts of Interest

The authors declare no conflicts of interest.

## Data Availability

The data that support the findings of this study are available on request from the corresponding author.
